# Antioxidant therapy against TGF‐β/SMAD pathway involved in organ fibrosis

**DOI:** 10.1111/jcmm.18052

**Published:** 2023-12-02

**Authors:** Soudeh Ghafouri‐Fard, Arian Askari, Hamed Shoorei, Mohammad Seify, Yeganeh Koohestanidehaghi, Bashdar Mahmud Hussen, Mohammad Taheri, Majid Samsami

**Affiliations:** ^1^ Department of Medical Genetics, School of Medicine Shahid Beheshti University of Medical Sciences Tehran Iran; ^2^ Phytochemistry Research Center Shahid Beheshti University of Medical Sciences Tehran Iran; ^3^ Cellular and Molecular Research Center Birjand University of Medical Sciences Birjand Iran; ^4^ Clinical Research Development Unit of Tabriz Valiasr Hospital Tabriz University of Medical Sciences Tabriz Iran; ^5^ Research and Clinical Center for Infertility, Yazd Reproductive Sciences Institute Shahid Sadoughi University of Medical Sciences Yazd Iran; ^6^ Department of Clinical Analysis, College of Pharmacy Hawler Medical University Erbil Iraq; ^7^ Institute of Human Genetics Jena University Hospital Jena Germany; ^8^ Urology and Nephrology Research Center Shahid Beheshti University of Medical Sciences Tehran Iran; ^9^ Cancer Research Center, Loghman Hakim Hospital Shahid Beheshti University of Medical Sciences Tehran Iran

**Keywords:** antioxidants, extracellular matrix, organ fibrosis, oxidative stress, TGF‐β/SMAD pathway

## Abstract

Fibrosis refers to excessive build‐up of scar tissue and extracellular matrix components in different organs. In recent years, it has been revealed that different cytokines and chemokines, especially Transforming growth factor beta (TGF‐β) is involved in the pathogenesis of fibrosis. It has been shown that TGF‐β is upregulated in fibrotic tissues, and contributes to fibrosis by mediating pathways that are related to matrix preservation and fibroblasts differentiation. There is no doubt that antioxidants protect against different inflammatory conditions by reversing the effects of nitrogen, oxygen and sulfur‐based reactive elements. Oxidative stress has a direct impact on chronic inflammation, and as results, prolonged inflammation ultimately results in fibrosis. Different types of antioxidants, in the forms of vitamins, natural compounds or synthetic ones, have been proven to be beneficial in the protection against fibrotic conditions both in vitro and in vivo. In this study, we reviewed the role of different compounds with antioxidant activity in induction or inhibition of TGF‐β/SMAD signalling pathway, with regard to different fibrotic conditions such as gastro‐intestinal fibrosis, cardiac fibrosis, pulmonary fibrosis, skin fibrosis, renal fibrosis and also some rare cases of fibrosis, both in animal models and cell lines.

## 1 INTRODUCTION

Fibrotic diseases account for 45% of mortality in the United States.^1^ Fibrosis usually happens as a result of disruption in body's natural ability to repair scars and damages.^2^ In fact, fibrosis is a phenomenon described by extreme accumulation of collagen and other extracellular matrix (ECM) components.^3^ Subsequent to an injury or scar, the affected area and immune cells, especially macrophages, secrete different kinds of chemokines and cytokines, as well as signalling proteins, such as TGF‐β.^4^ Consequently, secretion of these proteins causes an increased rate of proliferation and also migration of fibroblasts to the affected area. After migration, fibroblasts are usually differentiated to myofibroblasts in order to repair damages more efficiently. Chronic inflammation and exposure to harmful substances cause over‐healing and excessive repair of damaged tissues and ultimately fibrosis.^5^ This phenomenon is lethal if happens in vital organs, including lung, liver, heart and kidney.^5^

Another mechanism which is involved in the fibrosis is epithelial‐mesenchymal transition (EMT). Through this process, cells lose their epithelial characteristics, including apical–basal polarity and stable intercellular junction, and gain mesenchymal features such as cytoskeletal and morphological rearrangements and fibroblast‐like gene signature.^6^

Transforming growth factor beta (TGF‐β) is a protein ligand that activates a cascade of reactions by binding to serine threonine kinase receptors.^7^ TGF‐β is involved in different cellular mechanisms. For instance, it impedes proliferation and regulates ECM and collagen synthesis.^8^ Increased amount of ECM is a characteristic of most inflammatory diseases.^9^ Overexpression of TGF‐β causes tissue fibrosis and scars in different organs.^10^ Upon binding of TGF‐β, a group of transcription factors, called SMADs are phosphorylated. SMADs are the main signal transducers of the TGF‐β family. After phosphorylation, they migrate to nucleus and in accordance with other factors, SMADs contribute to activation or inhibition of different genes.^11^ TGF‐β is involved in cell growth and development. Moreover, it regulates inflammatory responses and preservation of resistance mechanisms against inflammation. Another aspect of TGF‐β functions which is associated with human disorders is remodelling and repair process. The latter contributes to the angiogenesis and tissue regeneration.^12^

Antioxidants are agents that could oppose adverse effects of oxidation from intracellular compounds.^13^ Based on their mechanism of action, antioxidants are divided into three groups, including primary, secondary and tertiary ones. Primary antioxidants act free radical scavengers; secondary antioxidants retard chain initiation; and tertiary ones mainly repair damaged biomolecules. A generalized function of almost all types of antioxidants is reversing the effects of ROS in cells.^14^ Both endogenous and exogenous antioxidants have essential roles in maintaining an optimum cellular function. Reactive oxygen species (ROS) and different nitrogen and sulfur‐based compounds, which are normally generated in cells as a result of different interactions and reactions, are potentially harmful substances and could damage cells at DNA, lipid, protein, carbohydrate and other levels.^15^ Thus, a proper and balanced diet enriched with antioxidants could actually reverse detrimental effects of these agents. An important instance of endogenous cellular antioxidants is glutathione, which is composed of three amino acids, including glutamine, cysteine and glycine. This antioxidant confronts adverse effects of hydroperoxide and other peroxides, with the help of glutathione peroxidase (GPX).^16^ At molecular level, antioxidants reduce the cellular levels of free radicals through inhibition of activity or expression of free radical producing enzymes, including NAD(P)H oxidase and xanthine oxidase (XO). Besides, antioxidants can enhance the activity and expression of antioxidant enzymes, namely superoxide dismutase (SOD), catalase (CAT) and GPX.^17^ Vitamin C (ascorbic acid) is a great example of exogenous agents, which helps with iron absorption and reversing free radical effects.^18^ In recent years, antioxidants are widely used in order to prevent and treat different human disorders, especially in animal models. Some of them, including edaravone and Nacetylcysteine gained the right permission for clinical use.^19^ The road ahead of using these agents as therapeutic elements is long, but daily experiments are conducted on different cell lines and animal models, in order to potentiate their therapeutic properties.

In this study, we aim at reviewing the role of different kinds of antioxidants as therapeutic agents in organ fibrosis, with regard to TGF‐β/SMAD pathway as the main target of antioxidants.

## 2 OXIDATIVE STRESS AND ITS ROLE IN ORGAN FIBROSIS

Oxidative stress is a condition that arises when there is an imbalance between the production of ROS and antioxidant defence mechanisms in the body.^20^ ROS are highly reactive molecules that are generated as a byproduct of normal cellular metabolism, but they can also be produced in response to external factors such as toxins, radiation and infections.^21^, ^22^ Oxidative stress can cause damage to proteins, lipids, and DNA, leading to cellular dysfunction and tissue damage.^23^

ROS can stimulate the activation of fibroblasts,^24^ which are cells responsible for the production of ECM proteins such as collagen, fibronectin and elastin.^25^ In addition, ROS can also stimulate the release of pro‐inflammatory cytokines and growth factors that promote fibroblast activation and ECM deposition.^26^ Moreover, ROS can inhibit the activity of matrix metalloproteinases (MMPs), which are enzymes that degrade ECM proteins, thereby impairing the process of ECM turnover and promoting fibrosis.^27^, ^28^

In the context of organ fibrosis, oxidative stress plays a critical role in the initiation and progression of the fibrotic process,^29^ which is characterized by the excessive deposition of ECM proteins, and leads to the progressive loss of organ function.^30^ Several studies have shown that oxidative stress can promote fibrosis by inducing the activation of fibro‐genic cells such as fibroblasts and myofibroblasts.^31^

Oxidative stress can activate several signalling pathways that contribute to fibrosis, including transforming growth factor‐beta (TGF‐β).^32^ TGF‐β is a potent inducer of ECM synthesis and is known to promote the differentiation of fibroblasts into myofibroblasts, which are the primary producers of ECM proteins.^33^ Moreover, oxidative stress can also contribute to fibrosis by impairing the function of antioxidant defence mechanisms.^34^ Studies have shown that antioxidant levels are reduced in fibrotic organs, and this reduction is associated with increased oxidative stress and fibrosis.^35^

Overall, oxidative stress plays a critical role in the pathogenesis of organ fibrosis by promoting fibro‐genic signalling pathways and impairing antioxidant defence mechanisms. Therefore, targeting oxidative stress may represent a potential therapeutic strategy for the prevention and treatment of fibrotic diseases.

## 3 ANTIOXIDANTS AS A MODULATOR OF TGF‐Β/ SMAD PATHWAY IN DIFFERENT ORGAN FIBROSIS

The TGF‐β/SMAD signalling pathway plays a key role in the development and progression of organ fibrosis by promoting fibroblast activation and ECM protein accumulation.^36^ Antioxidants have been proposed as potential modulators of the TGF‐β/SMAD pathway in various organ fibrosis conditions. Here are some examples:

### 3.1 Gastro‐intestinal fibrosis

Liver fibrosis refers to accumulation of ECM components, especially collagen in the liver, which ultimately results in liver failure or cirrhosis. Most of the time, the treatment includes liver transplant.^37^ Different kinds of fibrogenic cytokines are involved in liver fibrosis, and TGF‐β is one of them.^38^

In this section, we inquire role of different antioxidants as regulators of TGF‐β/ SMAD in gastro‐intestinal fibrosis, with an especial focus on liver fibrosis (Table 1).

**TABLE 1:** Role of different antioxidants as regulators of TGF‐β/ SMAD in treatment of liver fibrosis (LF).

Treatment	Model of study	Cell lines	TGF‐β/SMAD	Targets	Observations	Ref
Vitamin E	C57BL6/J mice	–	Inhibit	COX‐2, NF‐kappaB, Bcl‐2, Bax MMP2	Vitamin E ameliorated oxidative stress, hepatic apoptosis, and necroinflammation	^39^
N‐terminal latency‐associated peptide (LAP) and Truncated LAP (tLAP)	Male C57BL/6 mice; LAP and tLAP (60 μg/mouse)	HSC‐T6, AML12 0–60 μg/mL	Inhibit	α‐SMA, FN, Col‐I E‐cadherin, Smad‐2	LAP and tLAP could alleviate CCL4‐induced LF	^40^
LAB strains (Weissella cibaria, Lactobacillus brevis, Lactiplantibacillus plantarum)	–	LX‐2 5 × 10^7^ cells/mL	Inhibit	Col1A1, α‐SMA, MMP‐2, p38, p62, TIMP‐1/2, ICAM‐1, IL‐1β, TNF‐α, TRAF6, Smad‐2/3/7, ERK, ATG5, LC3I/II, Akt/mTOR	In hepatic stellate cells, treatment with probiotics could alleviate LF by suppressing the mentioned pathway and autophagy	^41^
Hepatocyte growth factor (HGF) + adipose‐derived stem cells (ADSCs)	CD1 mice HGF (150 μg/kg) + ADSCs (10^4^ cells/μL)	Hepatic stellate cells; HGF (50 ng/mL) + ADSCs (10^4^ cells/cm^2^)	Inhibit	Col‐I, α‐SMA, Smad‐2/3/7	Treatment with ADSCs + HGF could act against CCL4‐induced LF	^42^
Ferulic acid (FA)	Male Wistar rats; FA (10 mg/kg)	LX‐2 FA (0–50 μM)	Inhibit	α‐SMA, FN, Col‐I, Smad‐2/3, p38, JNK	Treatment with FA could act against CCl4‐induced LF. It acts through reduction of phosphorylation of SMAD2/3 and reversing SMAD4 nuclear translocation	^43^
Ligustroflavone (LIF)	C57BL/6J mice; LIF (5 and 20 mg/kg)	LX‐2 LIF (25 μmol/L)	Inhibit	α‐SMA, COL1A1, Vimentin, E‐cadherin, Smad‐2/3/4	Treatment with IF could act against CCL4‐induced LF	^44^
vinegar Curcuma wenyujin (VCW), Curcuma wenyujin Y.H. (CW)	Male SD rats, VCW (0.95 and 1.9 g/kg), CW (0.95 and 1.9 g/kg)	HSC‐T6 CW and CW (5%–20% serum)	Inhibit	Smad‐2/3/7, MMP‐2, TIMP‐1, PAI‐1	Treatment with CW and VCW could act against LF	^45^
Propylene glycol alginate sodium sulphate (PSS)	Male C57 mice; PSS (12.5, 25, 50 mg/kg)	LX‐2 PSS (0–15 μg/mL)	Inhibit	Col‐I, α‐SMA, MMP‐2, TIMP‐1, Smad‐2/3, JAK2/STAT3, MMP‐2/1	Treatment with PSS could act against CCL4‐induced LF	^46^
Honokiol	Male SD rats, honokiol (10 mg/kg(	–	Inhibit	α‐SMA, Smad‐2/3	Treatment with Honokiol could act against LF induced by concanavalin‐A	^47^
Gooseberry anthocyanins (GA)	Male Kunming mice; GA (20 and 40 mg/kg)	–	Inhibit	α‐SMA, Col‐I, Samd‐2	Treatment with GA could act against CCL4‐induced LF hway	^48^
Copper nanoparticles (Cu‐NPs)	Male SD rats; Cu NPs (100, 200, 400 mg/kg)	Mononuclear Cells (MNCs)	Induce	α‐SMA, Col1A2, Col‐III, Smad‐2/3, MAPK, Akt/FoxO3	Treatment with Cu‐NPs could induce LF and hepatic damage	^49^
Puerarin (PUR)	Male SD; PUR (100 mg/kg)	–	Inhibit	α‐SMA, Col1A1, Smad‐2/3	Treatment with PUR could act against LF induced by STZ	^50^
Simvastatin (Sim), BM‐MSCs	Male SD rats; Sim (10 mg/kg) + BM‐MSCs (10^6^)	HSCs; (BM‐MSCs 1:1 ratio co‐culture)	Inhibit	α‐SMA, Smad3, Col‐I	Treatment with Sim + MSCs could act against LF induced by thioacetamide (TAA)	^51^
Tectona Grandis (TG)	C57BL/6 mice, TG (50–200 mg/kg)	Vero and HepG2; TG (0–200 μg/mL)	Inhibit	α‐SMA, Col‐Iα, TIMP1, MMP3, Smad‐2/7	Treatment with TG could act against CCL4‐induced LF by elevating the ratio of MMP3/TIMP1	^52^
Morin	Male Wistar rats; Morin (50 mg/kg)	LX‐2 Morin (50 μM)	Inhibit	Col‐I/III, Smad2/3, MMP‐1/2/9, Wnt/β‐catenin, Hippo/Yap	Treatment with Morin could act against liver fibrosis induced by diethylnitrosamine by inhibiting the mentioned pathway	^53^
Paeoniflorin (PF)	Male SD rats; PF (50, 100, 200 mg/kg)	–	Inhibit	α‐SMA, Col1a1, Smad‐2/3/7	Treatment with PF could act against CCL4‐induced LF	^54^
Casticin	Male mice; Casticin (20 mg/kg)	LX 2 cells; Casticin (0–40 μM)	Inhibit	α‐SMA, Col1α1, TIMP1/2 Smad3/2 MMP‐2/9	Treatment with Casticin could act against CCL4‐induced LF by elevating the ratio of TIMP1/2 and MMP‐2/9	^55^
Mirtazapine	Male albino Swiss; mirtazapine (5, 10 mg/kg)	–	Inhibit	α‐SMA, Smad‐3, Procollagen‐I ERK1/2	Treatment with mirtazapine could act against the progression of liver fibrosis induced by thioacetamide (TAA) by inhibiting the mentioned pathway	^56^
NAOs	Male ICR mice; NAOs (0.5 mg/kg)	HSCs, LX‐2 NAOs (0–1 mg/mL)	Inhibit	α‐SMA, COL1A1 Smad‐2/3	Treatment with NAOs could act against CCL4‐induced LF	^57^
Schisandrin B (Scheme B)	Male Wistar rats; Scheme B (25 or 50 mg/kg)	HSC‐T6 5–30 μM	Inhibit	α‐SMA, IL‐1β, Smad‐2/3, HO‐1 Nrf‐2, NQO1, COL1/3A1, ERK	Treatment with Scheme B could act against CCL4‐induced LF by regulating the Nrf2‐ARE pathway	^58^
Grifola Frondosa (GFP)	Male SD rats; GFP (50, 100, 200 mg/kg)	–	Inhibit	Smad‐2/3/4, miR‐145, miR‐146a, PAI‐1	Treatment with GFP could act against CCL4‐induced LF	^59^
Praziquantel (PZQ)	Female BALB/c mice; PZQ (300 mg/kg)	LX‐2, MES13, NIH3T3; PZQ (20 and 30 μg/mL)	Inhibit	α‐SMA, Col1A1, Smad‐3/4/7	Treatment with PZQ could act against CCL4‐induced LF by elevating Smad‐7	^60^
Graptopetalum paraguayense (GP, or HH‐F3)	Wistar rats; HH‐F3 (0.05 and 0.15 g/kg)	LX‐2, HSC‐T6 HH‐F3 (5, 10, 15 μg)	Inhibit	α‐SMA, Col‐I/III, Elastin, TIMP1, Smad‐2	Treatment with HH‐F3 could act CCL4‐induced LF	^61^
CCM111	Male CD1 (ICR) mice; CCM111 (20 and 100 mg/kg)	HSC‐T6; CM111 (0–160 mg/mL)	Inhibit	α‐SMA, MMP‐2, Smad‐2/33, Wnt, β‐catenin	Treatment with CCM111 could act against CCL4‐induced LF	^62^
Alogliptin (ALO)	Male C57BL/6 J mice; ALO (20 mg/kg)	LX‐2 ALO (0–80 μM)	Inhibit	α‐SMA, FN, Col‐I, Smad‐2/3, Akt	Treatment with ALO could act against CCL4‐induced LF	^63^
Isorhamnetin (IsoR)	Male ICR mice; IsoR (10 and 30 mg/kg)	LX‐2; IsoR (50 or 100 μM)	Inhibit	α‐SMA, Col1A1, Smad‐2/3	Treatment with IsoR could act against CCL4‐induced LF	^64^
Genistein	Male Wistar rats; Genistein (5 mg/kg)	–	Inhibit	Smad‐2/3/7, Col‐I/III	In an animal model of FHF induced by D‐galactosamine (D‐GalN), treatment with genistein via suppressing the mentioned pathway could act against liver damage	^65^

**FIGURE 1:**
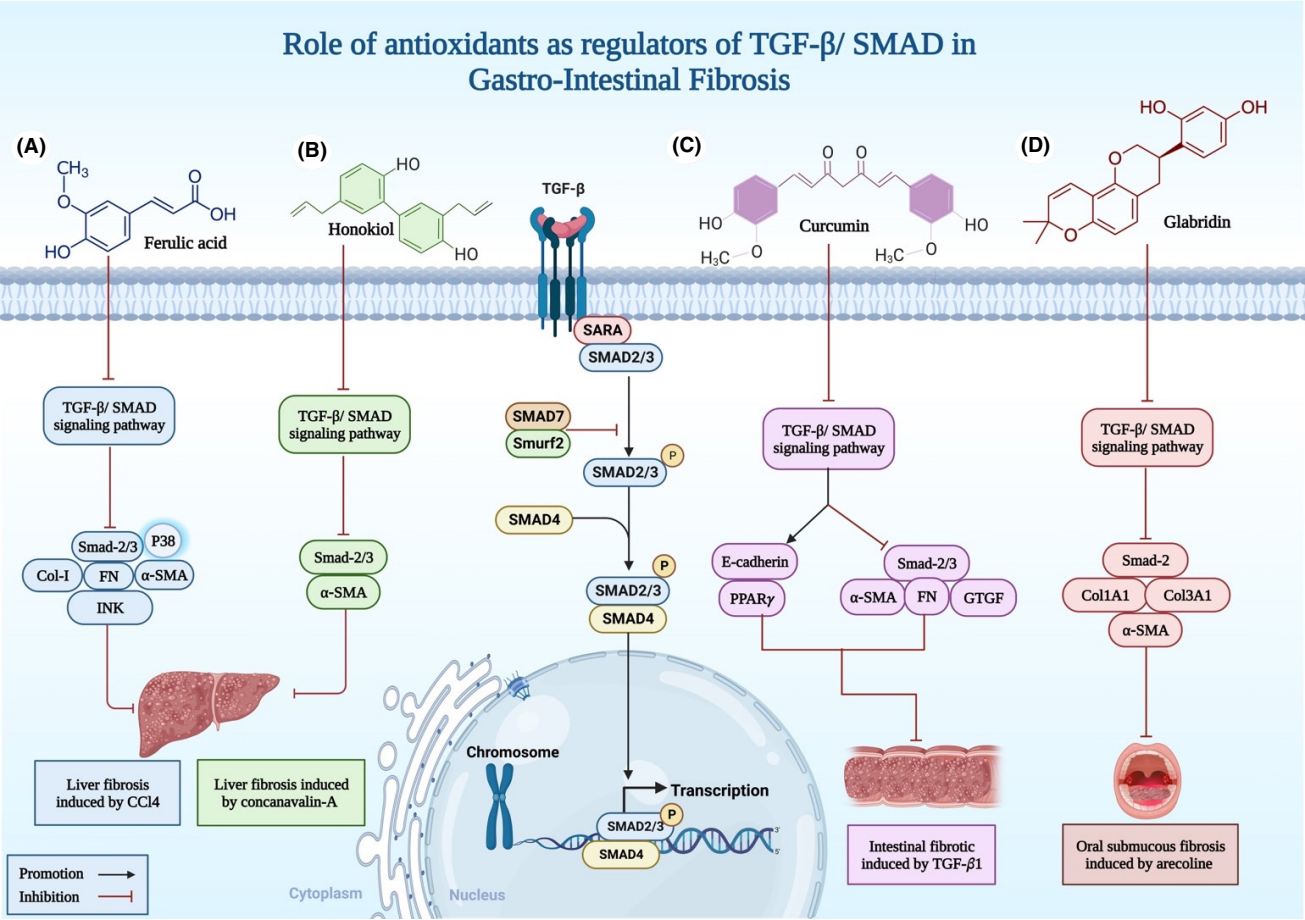
Schematic diagram illustrating the mechanism of action for antioxidants in regulating TGF‐β/SMAD signalling pathway and preventing Gastro‐Intestinal Fibrosis.

As shown in Table 1, most of the studied agents, had an inhibitory role on TGF‐β/ SMAD pathway. As it was mentioned before, TGF‐β/SMAD stimulation contributes to increased rate of ECM synthesis. Ferulic acid (FA) is a plant derived antioxidant and a free radical scavenger (a primary antioxidant) with proven beneficial roles in different conditions, especially cancer and inflammatory diseases.^66^ In a study conducted by Mao mu and colleges in 2018, it was demonstrated that treatment with FA could reverse the effects of carbon tetrachloride (CCl4) induced liver fibrosis.^43^ More specifically, treatment with TGF‐β causes an overexpression of α SMA, FN, Col‐I, Smad‐2/3, p38 and JNK. Treatment of LX‐2 cells with 30 μM (50 μM was cytotoxic) of FA reversed the effects of TGF‐β in these cells^43^ (Figure 1A).

Honokiol is an antioxidant with a poly‐phenolic nature, mostly derived from magnolol and root bark, with protective properties against neural damage, anxiety and a variety of cognitive disorders.^67^ It has been shown that treatment of male SD rats with concanavalin‐A, elevated alpha smooth muscle actin (α‐SMA) levels in liver, which is a fibrosis indicator in most cases.^47^ After treatment with Honokiol for 4 weeks, reduced expression of (α‐SMA) was seen.^47^ Additionally, it was documented that Honokiol acts as TGF‐β/SMAD/MAPK inhibitor and protects against liver fibrosis^47^ (Figure 1B).

In the category of flavonoids, Isorhamnetin (IsoR) belongs to class of Flavonols and is mostly found in *Hippophae rhamnoides L* fruits.^68^ IsoR play a role in protection against various conditions, such as cardiac and cerebral related complications.^68^ This agent could reverse liver fibrosis with a similar mechanism as FA.^64^

Based on a study performed by Tengfei Liu and colleges, it was shown that copper‐based nanoparticles could act as antioxidant by scavenging ROS and reducing inflammation both in vitro and in vivo.^69^ Interestingly, their antioxidant property is at stake in case of protection against liver fibrosis. In a concise study, it was proved that treatment of Mononuclear Cells (MNCs) with copper‐based nanoparticles, induces TGF‐β/SMAD pathway and subsequent elevation of ECM compounds occurs, leading to liver fibrosis. Copper‐based nanoparticles are the only exception of antioxidants that could activate TGF‐β pathway and contribute to formation of fibrotic tissue.

The result of inflammatory bowel disease (IBD) in both forms, including ulcerative colitis (UC) and Crohn's disease (CD), is mostly intestinal fibrosis. Similar to liver fibrosis, excessive amount of scar tissue and over repaired damage is the cause.^70^ Two independent studies confirmed the role of antioxidants against intestinal fibrosis. Firstly, it was demonstrated that treatment of intestinal epithelial cells (IEC‐6) with Curcumin (CUR), had two significant results: (1). inhibition of TGF‐β1 induced SMAD pathway and downregulation of α‐SMA and (2). Increased expression of peroxisome proliferator‐activated receptor γ (PPARγ) and its nuclear localization, which inhibits EMT and protects against fibrosis^71^ (Figure 1C).

Another study also confirmed the role of Calycosin (CA), which is a component of astragalus membranaceus, in fighting against intestinal fibrosis.^72^ This protection is done through the same mechanism as CUR, but the different is that CA does not interfere with EMT, instead, it elevates Smad7 expression, which is an inhibitory Smad and inhibits TGF‐β signalling.^72^ Further, The polyphenol glabridin, which is derived from Glycurrhiza glabra (licorice) roots, has been utilized in traditional medicine and is known to have a variety of biological actions, including anti‐inflammatory, antioxidant, and anti‐fungal effects.^73^ One of the oral conditions that may be malignant is known as oral submucous fibrosis (OSF). By inhibiting TGF/smad signalling, glabridin prevents myofibroblast activation in human fibrotic buccal mucosal fibroblasts^73^ (Figure 1D).

Table 2 shows the effects of different antioxidants in regulation of TGF‐β/ SMAD pathway in other gastrointestinal disorders.

**TABLE 2:** The effects of different antioxidants in regulation of TGF‐β/ SMAD pathway in other gastrointestinal disorders.

Diseases	Treatment	Model of Study	Cell lines	TGF‐β/SMAD	Targets	Observations	Ref
Peritoneal dialysis (PD)	Parthenolide (PTL)	C57BL/6J mice; PTL (12.5–50 mg/kg)	HMrSV5 0–5 μM	Inhibit	E‐cadherin, Smad‐2/3, ERK, FN, Col‐I	Treatment with PTL by inhibiting the mentioned pathway could act against peritoneal fibrosis	^74^
Peritoneal fibrosis	Empagliflozin (EMP)	Male C57BL/6 mice, EMP (6 mg/kg)	Peritoneal mesothelial cells (HPMCs); EMP (1 μM)	Inhibit	α‐SMA, Col‐I, E‐cadherin, Smad‐3	Treatment with EMP could act against peritoneal fibrosis	^75^
Peritoneal fibrosis	Tanshinone IIA (T‐IIA)	–	HPMCs; T‐IIA (50 and 100 μM)	Inhibit	α‐SMA, FN, Col‐I E/N‐cadherin MMP‐2/9 Smad‐2/7	Treatment with T‐IIA could act against peritoneal fibrosis	^76^
Chronic pancreatitis (CP)	Piperine	Female C57BL/6 mice; piperine (1–10 mg/kg)	‐	Inhibit	α‐SMA, FN‐1, Col‐I/III, Smad‐2/3, IL‐6, TNF‐α, Ll‐1β	In an animal model of CP, treatment with piperine could act against fibrosis severity	^77^
Pancreatic fibrosis	Scoparone	Male SD rats; Scoparone)60 and 30 mg/kg (	PSCs; Scoparone (0.1–04 mmol/L)	Inhibit	α‐SMA, Col‐I, E‐cadherin, Vimentin, Smad‐2/3/7	Treatment with Scoparone could act against pancreatic fibrosis induced by dibutyltin dichloride (DBTC)	^78^
Intestinal fibrosis	Curcumin (CUR)	SD rats; CUR (50, 100, 200 mg/kg)	IEC‐6 CUR (2.5–10 μM)	Inhibit	α‐SMA, FN, E‐cadherin, smad‐2/3, PPARγ, CTGF	Treatment with CUR could act against intestinal fibrotic induced by TGF‐β1 via inhibiting the mentioned and EMT pathways	^71^
Intestinal fibrosis	Calycosin (CA)	–	CCD‐18Co CA (0–800 μmol/L)	Inhibit	α‐SMA, Col‐I, Smad‐2/3/4/7	Treatment with CA could act against TGF‐β1‐induced intestinal fibrosis	^72^
Oral submucous fibrosis (OSF)	Glabridin (GLA)	–	Buccal Mucosa Fibroblasts (fBMFs1/2); Glabridin (0–20 μM)	Inhibit	α‐SMA, Col1A1, Col3A1, Smad‐2	Treatment with GLA could act against arecoline‐induced OSF	^73^

### 3.2 Lung fibrosis

Pulmonary or lung fibrosis (PF) refers to progressive lung scarring with potential life‐threatening properties.^79^ There has been an incremental trend In pulmonary fibrosis in recent years.^80^ Different risk factors such as smoking, exposure to harmful substances and diabetes could ultimately lead to fibrosis of the lungs.^81^

Induction of TGF‐β/SMAD pathway by antioxidants in PF was seen in multiple researches. In 2016, it was observed that treatment of A549 cells with Bleomycin (BLM), a cancer related antibiotic,^82^ could induce TGF‐β/ SMAD pathway, and this was concluded based on lower levels of E‐cadherin and elevated levels of vimentin after treatment with BLM.^83^ Additionally, respiratory exposure to nanoparticulate titanium dioxide (nano‐TiO2) has proven to be detrimental to lungs by elevating expression of proteins such as TGF‐β1 and HIF‐1α and conversely downregulating phosphorylated glycogen synthase kinase‐3β (p‐GSK‐3β).^84^ It is evident that Interleukin‐19 (IL‐19) has great anti‐inflammatory properties, especially in vascular diseases,^85^ yet, this function is not evident in the case of PF. Combination of wet lab and dry lab techniques has led to this understanding that IL‐19 is elevated in fibrotic lung (^86^). It was demonstrated that this cytokine has adverse effects on idiopathic pulmonary fibrosis (IPF) both in vitro and in vivo (^86^). Interestingly, all of the mentioned effects are directly reversed by LY2109761, which inhibits TGF‐β/ SMAD signalling pathway (^86^).

It is worth mentioning that certain vitamins, such as vitamin C and D act as antioxidants. In relation to respiratory fibrosis and inflammation, a great number of studies are about asthma and its complications. Research has shown that ovalbumin (OVA) induced asthma in murine models is reversed by intraperitoneal injection of vitamin D in its active form, 1, 25‐dihydroxyvitamin D3.^87^ It was shown that vitamin D activates Nrf2/HO‐1 pathway, which itself activates gene expression of heme oxygenase‐1 (HO‐1), an anti‐oxidative stress agent.^88^ Moreover, vitamin D acts as a TGF‐β/SMAD inhibitor in asthmatic airway.^87^

**FIGURE 2:**
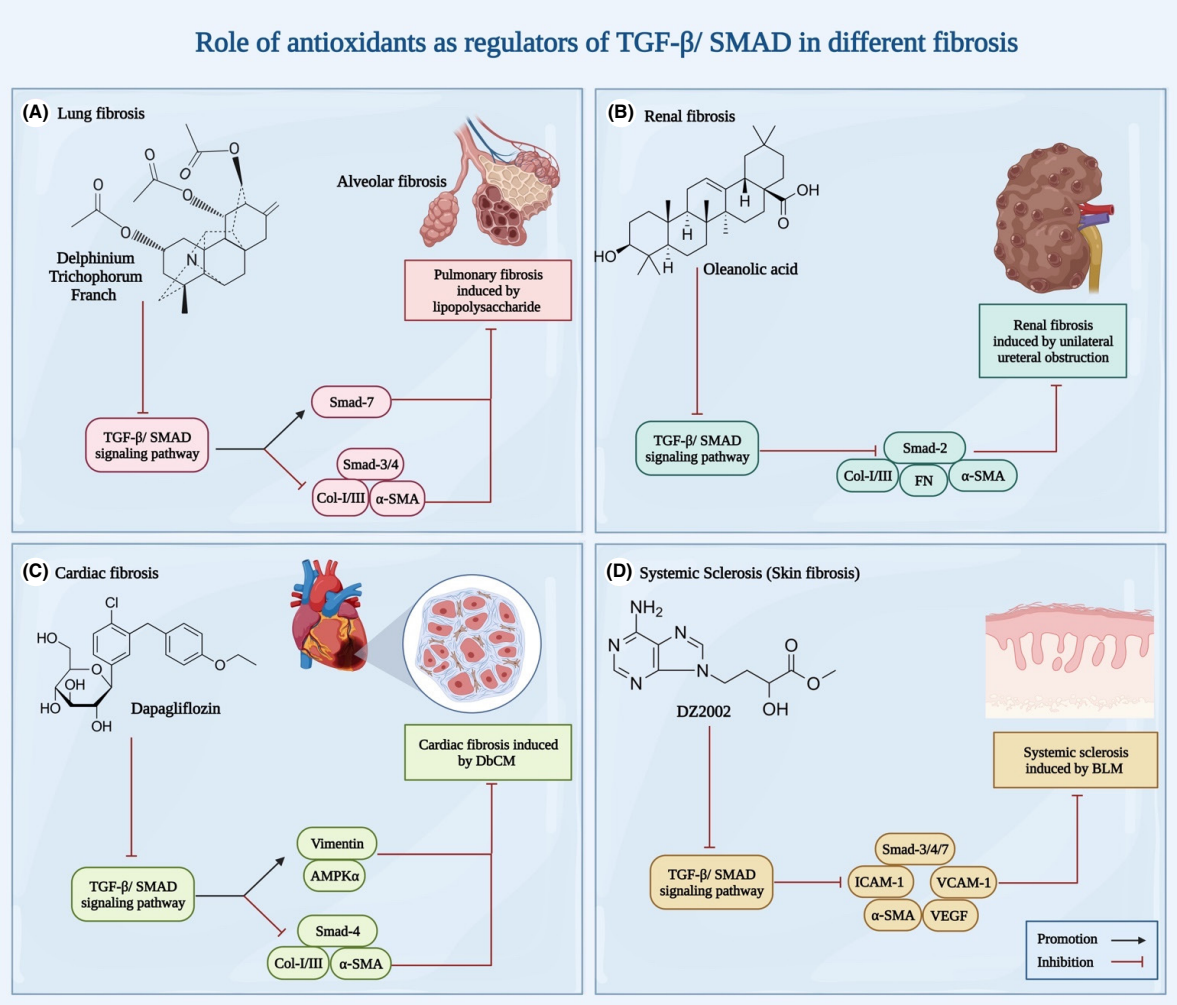
Antioxidants as potential therapeutic agents: a visual overview of their role in modulating TGF‐β/SMAD pathways across different fibrosis types.

As it is evident in Table 2, BLM and lipopolysaccharides (LPS) are used for induction of pulmonary fibrosis, and in most cases, treatment with different substance reverses this process by inhibiting TGF‐β/SMAD and other inflammatory pathways. On the most recent studies centered around this has been done by Yao y and colleges in 2022.^89^ Till this day, more than 1500 of Diterpenoid alkaloids (DAs) have been extracted from different plants. Some of these alkaloids have shown cytotoxic effects on cancer cells.^90^ By implanting molecular docking, It was shown DAs extracted from Delphinium trichophorum (DTF), especially DTF1 and DFT2, hamper TGF‐β/ SMAD pathway and protect against PF in various ways, including interaction with TGFB receptor 1 (TGFBR1) and covering it, downregulating TGF‐β1 and α‐SMA, and restoring Smad7, the inhibitory Smad^89^ (Figure 2A).

Table 3 summarizes treatment of PF models and cell lines with different antioxidants and their impact on TGF‐β/ SMAD pathway.

**TABLE 3:** Lung fibrosis.

Diseases	Treatment	Model of Study	Cell Lines	TGF‐β/ SMAD	Targets	Observations	Ref
Pulmonary fibrosis (PF)	Bleomycin (BLM)	–	A549 BLM (0–50 μg/mL)	Induce	α‐SMA, FGFR2, E‐cadherin, Vimentin, Smad‐2/3	Treatment with BLM via inducing EMT and TGF‐β/Smad pathway could lead to severe PF	^83^
PF	Trichocarboline	–	HFL1 0–10 μM	Inhibit	FN, α‐SMA, PCNA	Treatment with trichocarboline could act against pulmonary fibrosis	^91^
PF	Delphinium Trichophorum Franch (DTF)	–	HFL‐1, 3 T6 DTF (0–50 μM)	Inhibit	α‐SMA, Col‐I/III, Smad‐3/4/7	Treatment with diterpenoid alkaloids extracted from DTF could act against lipopolysaccharide (LPS)‐induced PF	^89^
PF	Tanshinone IIA (Tan IIA)	Wistar rats; Tan IIA (25 mg/kg)	Pulmonary Fibroblasts Tan IIA (53, 160, 480 μg/mL)	Inhibit	Smad‐2/3/7, Col‐I/III, α‐SMA,	Treatment with Tan IIA could act against silica induced PF	^92^
PF	Ferulic Acid (FA)	Male Swiss albino mice; FA (100 and 300 mg/kg)	–	Inhibit	Col‐I, Slug, α‐SMA E‐cadherin, Vimentin, Smad‐2/3	Treatment with FA could act against the progression of pulmonary fibrosis induced by crystalline silica by inhibiting the mentioned pathway	^93^
PF	Honokiol (HNK)	Swiss albino mice, HNK (1 and 3 mg/kg)	HFL1 HNK (0–3 μM)	Inhibit	E‐cadherin α‐SMA, FN, COL3A1, TIMP‐1 MMP‐7 Smad‐2/3	Treatment with HNK could act against BLM‐induced FP	^94^
PF	Nagilactone D (NLD)	Female C57BL/6J mice; NLD (10 mL/kg)	WI‐38 VA‐13, HLF‐1; NLD (5 ng/mL)	Inhibit	Col‐I/III, FN α‐SMA, CTGF Smad‐2/3	Treatment with NLD could act against BLM‐induced FP	^95^
PF	Rhapontin (RHA)	Male C57BL/6 mice; RHA (25, 50, 100 mg/kg)	THP‐1 RHA (0.1–100 μM)	Inhibit	α‐SMA, Col‐I Smad‐2/3, AMPK	Treatment with RHA could act against BLM‐induced FP	^96^
PF	Polydatin (PD)	Male SD rats; PD (100 mg/kg)	HFL‐1; PD (0–500 μM)	Inhibit	E‐cadherin, FN Smad‐2/3 Col‐I/III	Treatment with PD could act against BLM‐induced FP	^97^
PF	Peptide PD29	SD rats; PD29 (2.5, 5, 7.5 mg/kg)	A549 PD (0.1–100 μM)	Inhibit	α‐SMA, Col‐I E‐cadherin Smad‐2/3/7 MMP‐2/7/9/12, TIMP‐1/2	Treatment with PD29 could act against BLM‐induced FP	^98^
PF	Nanoparticulate titanium dioxide (nano‐TiO2)	ICR male mice; nano‐TiO2 (2.5, 5, 10 mg/kg)	–	Induce	α‐SMA, Col‐I/III, ILK, Smad‐2, TGF‐βR	nano‐TiO2 (respiratory exposure) could induce PF	^84^
PF	Nimbolide (NIM)	Male C57BL/6 mice; NIM (1 and 3 mg/kg)	–	Inhibit	α‐SMA, Col3A1, Col1A2, Smad‐2/3, N‐cadherin	Treatment with NIM could act against BLM‐induced FP	^99^
PF	Paeoniflorin (PAN)	Male ICR mice; PAN (25, 50, 100 mg/kg)	A549; PAN (0–30 μmol/L)	Inhibit	α‐SMA, Col‐I/III, Smad‐2/3, Snail, Slug, Twist, ZEB1/2, E‐cadherin, MAPK,	In pulmonary fibrosis, treatment with PAN could inhibit TGF‐β mediated EMT	^100^
PF	Salidroside	SD rats; Salidroside (50, 100, 200 mg/kg)	A549; Salidroside (50 μM)	Inhibit	α‐SMA, E/N‐cadherin, FN, Vimentin, Smad‐2/3, Nrf2, NQO1	Treatment with Salidroside could act against BLM‐induced FP	^101^
PF	Salvianolic Acid B (SAB)	C57BL/6 mice; SAB (10 mg/kg)	A549, MRC‐5, NIH/3 T3; SAB (50 μg/mL)	Inhibit	α‐SMA, FN, Col1A1/2, Ccl3A1, CTGF, PAI‐1, Smad‐3, ERK	Treatment with SAB could act against BLM‐induced FP	^102^
Idiopathic Pulmonary Fibrosis (IPF)	Nervilia Fordii Extract (NFE)	male SD rats; NFE (100–400 mg/kg)	3 T6 100–500 μg/mL	Inhibit	α‐SMA, ERK, Smad‐3/4/7	Treatment with NFE could act against BLM‐induced FP	^103^
IPF	IL‐19	C57BL/6 mice, 200 ng/kg	Primary mouse lung fibroblasts, HELF 0–200 ng/mL	Induce	α‐SMA, Col‐I, Smad‐2/3	Treatment with IL‐19 could increase IPF aggravation induced by BLM via triggering the mentioned pathway	(^86^)
IPF	Curdione (CUD)	Male C57BL/6 mice; CUD (100 mg/kg)	HPFs CUD (0–500 μM)	Inhibit	FN, Col‐I, α‐SMA, Smad‐2/3	Treatment with CUD could act against BLM‐induced IPF	^104^
IPF	Polydatin (POL)	male SD rats; POL (10, 40, 160 mg/kg)	A549 POL (10–120 μM),	Inhibit	Col‐I, α‐SMA, E‐cadherin, Smad‐2/3, ERK1/2	Treatment with POL could act against IPF induced by BLM via suppressing the ERK/TGF‐β/Smad pathway	^105^
IPF	Myricetin (MYR)	Male C57BL/6 mice; MYR (25,50, 100 mg/kg)	A549, ATCC, MLE12, Mlg, HFL1, NIH3T3; MYR (40 μM)	Inhibit	HSP90β, α‐SMA, Smad‐2	Treatment with MYR could act against BLM‐induced IPF	^106^
IPF	Biochanin‐A (BCA)	C57BL/6 J mice; BCA (10 and 5 mg/kg)	LL29 BCA (0–500 μM)	Inhibit	α‐SMA, FN1, COL1A1, COL3A1, Samd‐2/3/7, E‐cadherin	Treatment with BCA could act against IPF by suppressing the mentioned pathway	^107^
IPF	Evogliptin (EVP)	Male C57/BL mice; EVP (300 mg/kg)	Human lung fibroblasts; EVP (20 and 10 nM)	Inhibit	α‐SMA, Col‐I/III, Smad‐2/3	Treatment with EVP could act against BLM‐induced IPF	^108^
IPF	HYDAMTIQ (HYD)	Male C57BL/6 mice; HYD (1, 3, 10 mg/kg)	–	Inhibit	α‐SMA, COX‐2, Smad‐3,	Treatment with HYD could act against BLM‐induced IPF	^109^
Radiation‐Induced Pulmonary Fibrosis (RIPF)	Anastrozole	female Wistar rats; 0.003 mg/200 g of Anastrozole	–	Inhibit	PDGF, CTGF, Smad‐3, IL‐1β	Treatment with Anastrozole via inhibiting two pathways (TGF‐β/PDGF and TGF‐β/Smad) could act against RIPF	^110^
Radiation‐Induced Lung Fibrosis (RILF)	Thalidomide (THD)	Female C57BL/6 mice; THD (100 mg/kg)	THP‐1; THD (0.2 μmol/mL)	Inhibit	α‐SMA, FN, Col‐I, Smad‐3, Nrf2	Treatment with THD could act against RILF by inhibiting the mentioned pathway	^111^
Lung Allergic Inflammation	Vitex Negundo Leaf Extract (VNLE)	Balb/C mice; VNLE (150 and 300 mg/kg)	Macrophage; VNLE (0–200 μg/mL)	Inhibit	Smad‐2/3/4, PI3K/Akt, NF‐κB	Treatment with VNLE could act against fibrosis induced by OVA‐LPS (ovalbumin‐lipopolysaccharide)	^112^
Asthma	Vitamin D3	Female BALB/c mice; Vit D3 (100 ng)	–	Inhibit	α‐SMA, Smad‐2/3, Nrf2/HO‐1	Treatment with Vit D3 could act against ovalbumin (OVA)‐induced asthma and PF	^87^

### 3.3 Renal fibrosis

Renal fibrosis which manifests itself with tubulointerstitial fibrosis and glomerulosclerosis, is the end stage of chronic kidney disease.^113^ Similar to other fibrotic conditions, deposition of ECM in different areas of kidney, interrupts with its structure and function.^113^ It has been proved that TGF‐β plays a critical role in developing renal fibrosis, either by induction of apoptosis or EMT.^114^

Oleanolic acid (OA) is natural pentacyclic triterpenoid and is found in medicinal herbs and oils, specially olive oil.^115^ In an in vitro study performed in QZG cells in 2010, it was shown that OA has antioxidant activity by scavenging free radicals via increasing glutathione synthesis.^116^ Conveniently, it is demonstrated that oral administration of OA to male SD rats for 21 days, results is reduced expression of TGF‐β and its related receptors (I and II), as well as optimum levels of serum creatinine^117^ (Figure 2B).

While drugs may cause certain adverse effects, they can also have positive impacts. Losartan is a good example. Sold under the brand name ‘Cozaar’, losartan is widely used to treat high blood pressure via inhibiting angiotensin receptor.^118^ Alongside with its antihypertensive activity, a clinical trial conducted in 2013 has proven that treatment of patients undergoing haemodialysis with losartan not only increases thiol groups with antioxidant activity, but also reduces Oxidative stress index (OSI).^119^ The exact molecular mechanism of this phenomenon was discovered by studying mice models of Renal Interstitial Fibrosis (RIF). It was demonstrated that losartan treatment acts as an anti‐fibrosis agent by increasing inhibitory Smads, that is, Smad 7, and induction of TGF‐β receptor (I) breakdown.^120^

In case of high glucose exposure to simulate glucose‐mediated fibrosis, Human renal proximal tubule epithelial cell line (TH1) was treated with D‐glucose for a period of time.^121^ Subsequent treatment with melatonin (MEL), a sleep‐inducing hormone, was attentive. It was shown that MEL protects against high glucose induced renal fibrosis, by blocking TGF‐β expression and subsequent phosphorylation of Smads by it.^121^ Additionally, MEL increased Cellular prion protein (PrPC) expression via Akt activation.^121^ These interventions made by MEL ultimately results in protection against fibrosis.

Similar to liver and lung fibrosis, induction of TGF‐β/ SMAD pathway by antioxidants is also seen in renal fibrosis. Baicalin is a type of flavonoid compound and is derived from the root of Scutellaria baicalensis Georgi plant. This plant is a member of Lamiaceae family and is known for its medicinal properties.^122^ High dosage intake of Baicalin by Sprague–Dawley (SD) rats was shown to have detrimental effects on renal tissue.^123^ Based on dosage, Baicalin activates TGF‐β/Smad signalling pathway and contributes to renal fibrosis.^123^ Although it's not always the case, since a study conducted on mice models of RIF showed opposite results.^124^ It was revealed that baicalin acts as an anti‐fibrotic agent in renal fibrosis via suppressing the mentioned pathway.^124^

Table 4 summarizes treatment of renal fibrosis models and cell lines with different antioxidants and their subsequent impact on TGF‐β/ SMAD pathway.

**TABLE 4:** Kidney fibrosis.

Diseases	Treatment	Model of study	Cell Lines	TGF‐β/ SMAD	Targets	Observations	Ref
Renal fibrosis	4‐octyl itaconate (OI)	male SD rats; OI (1 or 10 mg/kg)	HK‐2; 1–100 μmol/L	Inhibit	NF‐κB, α‐SMA, FN, p65, IκBα, Smad‐2/3/7, LC‐3I/II	Treatment with OI via inhibiting the mentioned pathway, ROS, and autophagy could act against renal fibrosis	^125^
Renal fibrosis	Oleanolic acid (OA)	Male SD rats; OA (6 mg/kg)	–	Inhibit	α‐SMA, FN, Col‐I/III, Samd‐2	Treatment with OA could act against renal fibrosis induced by unilateral ureteral obstruction (UUO)	^117^
Renal fibrosis	Saroglitazar (SAR)	Male SD rats; SAR (3 mg/kg)	–	Inhibit	α‐SMA, MMP‐9, Smad‐3, PAI‐1	Treatment with SAR could act against renal fibrosis induced by UUO	^126^
Renal Fibrosis	Baicalin	SD rats; Baicalin)0–1600 mg/kg (	–	Induce	FN, Col‐I/IV, α‐SMA, Smad‐3, AMPK, CTGF	Baicalin promotes renal fibrosis by inducing the mentioned pathway	^123^
Renal fibrosis	Bardoxolone (BARD)	Male C56BL/6 mice; BARD (5 and 10 mg/kg (	MES 13; BARD (0.025–0.1 μM)	Inhibit	α‐SMA, FN, Smurf1/2, Smad‐2/3, Nrf2, Keap1	Treatment with BARD could act against renal fibrosis induced by aristolochic acid (AA)	^127^
Renal fibrosis	Lixisenatide (Lix)	Male SD rats; –	–	Inhibit	α‐SMA, VCAM‐1, ICAM‐1, Col‐I/IV Smad2/3/4/7,	Treatment with Lix could act against renal fibrosis induced by doxorubicin‐induced renal fibrosis	^128^
Renal fibrosis	Dalbergioidin (DAL)	mice; DAL (30 mg/kg)	–	Inhibit	α‐SMA, FN, Col‐III, Smad‐7, E‐cadherin	Treatment with DAL could act against renal fibrosis induced by doxorubicin by inhibiting the mentioned pathway	^129^
Renal fibrosis	Sorafenib	Male SD rats; Sorafenib (20, 40, 80 mg/kg)	NRK‐52E, Sorafenib (1, 5 and 10 μmol/L)	Inhibit	α‐SMA, E‐cadherin, Smad‐3	Treatment with sorafenib could act against renal fibrosis induced by UUO by inhibiting the mentioned pathway	^130^
Renal interstitial fibrosis (RIF)	Baicalin	male C57BL/6 mice; 10–40 mg/kg	Primary mouse fibroblasts, 50–150 μmol/L	Inhibit	α‐SMA, FN, Col‐I, Smad‐2/3, IL‐1β, IL‐6, TNF‐α	Treatment with baicalin could act against RIF by inhibiting the mentioned pathway	^124^
RIF	Losartan	Male C57BL/6 J mice, losartan (10–30 mg/kg)	–	Inhibit	α‐SMA, Smurf‐1/2, E‐cadherin, NOX4, Smad‐2/3/7	Treatment with losartan could act against RIF induced by UUO by inhibiting the mentioned pathway	^120^
RIF	Chrysophanol (CP)	C57BL/6 mice, CP (10, 20, 40 mg/kg)	HK‐2; CP (0–100 μM)	Inhibit	α‐SMA, FN, Col‐I/III, Samd‐2/3/4/7, Vimentin, TGF‐β‐RI/RII	Treatment with CP could act against RIF induced by UUO	^131^
RIF	Poricoic acid ZA (PZA)	–	HK‐2; PZA (0–100 μM)	Inhibit	α‐SMA, Vimentin, Col‐I/III, E‐cadherin, TGFβRI/RII, Smad‐2/3	Treatment with PZA could act against RIF induced by angiotensin II (ANGII) and TGF‐β1	^132^
Chronic Kidney Disease (CKD)	Corni Fructus (CF)	Male SD rats; CF (100 and 200 mg/kg)	–	Inhibit	α‐SMA, Col‐I, MMP2, NOX2, AMPK, NF‐kB, Smad‐2/3	Treatment with CF could act against renal fibrosis induced by unilateral ureteral obstruction (UUO)	^133^
CKD	Lindera aggregata ethanol extract (LEE), Lindera aggregata water extract (LWE)	Male SD rats; LWE (0.75–3.52 g/kg), LEE (0.88–3.52 g/kg)	HK‐2; LWE (100 μM) LEE (100 μM)	Inhibit	Smad‐2/3/7	Treatment with LEE and LWE could act against the progression of CKD and fibrosis induced by adenine	^134^
CKD	Melatonin (MEL)	–	TH1; Mel (1 μM)	Inhibit	FN, Col‐I, α‐SMA, E‐cadherin, Smad‐2/7,	Treatment with MEL could act against renal fibrosis induced by high glucose	^121^
CKD	Ganoderic Acid (GAA),	Male C57BL/6J mice; GAA (3.125, 12.5, 50 mg/kg)	HK‐2; GAA (0–100 μM)	Inhibit	E‐cadherin, Vimentin, α‐SMA, FN, Smad‐2/3/7, MAPK	Treatment with GAA could act against renal fibrosis induced by UUO by inhibiting the mentioned pathway and MAPK	^135^
CKD	Rhubarb extracts	Male SD rats; Rhubarb (200, 600, 80 mg/kg)	–	Inhibit	α‐SMA, Col‐I, E‐cadherin, FN, Vimentin, FSP1, Smad‐2/3/4/7 TGF‐βRI/RII	Treatment with rhubarb could act against renal fibrosis induced by adenine	^136^
CKD	Nootkatone (NTK)	Male Balb/C mice; NTK (5 and 10 mg/kg)	–	Inhibit	Col‐I/II, α‐SMA, FN, Smad‐2/3	Treatment with NTK could act against renal fibrosis induced by UUO	^137^
CKD	Nimbolide (NIM)	Male Swiss albino mice; NIM (0.3 and 1 mg/kg)	–	Inhibit	α‐SMA, Col‐I, E‐cadherin, FN, Vimentin, Slug, Smad‐2/3, CTGF	Treatment with NIM could act against renal fibrosis induced by UUO by inhibiting the mentioned pathway and Slug/EMT signalling	^138^
Chronic renal failure (CRF)	Acupuncture (Acup)	Male New Zealand White rabbits; Acup (−)	–	Inhibit	Smad‐3, ILK	Treatment with Acup could act against RIF induced by adenine	^139^
Renal fibrosis induced by diabetes	Astragalus Polysaccharides (AP)	Male SD rats; AP (25, 50, 100 mg/kg)	–	Inhibit	α‐SMA, Smad‐3 Col‐I/III/IV,	Treatment with AP could act against renal fibrosis	^140^
Renal fibrosis induced by diabetes	Melatonin (Mel)	Male SD rats; Mel (5, 15, 30 mg/kg)	–	Inhibit	Smad‐2/3, Wnt4, β‐catenin, EGFR	Treatment with Mel could act against STZ‐induced renal fibrosis	^141^
Renal fibrosis induced by diabetes	Dencichine (DE)	Male SD rats; DE (60,80,160 mg/kg)	HBZY‐1; DE (1.0 × 10^−5^/ 10^−4^/10^−3^ M)	Inhibit	Smad‐2/3/7, MMP‐9, TIMP‐1, FN, Col‐I/IV	Treatment with DE could act against the progression of renal fibrosis induced by STZ	^142^

### 3.4 Cardiac fibrosis

Cardiac fibrosis is seen in different cardiomyopathies, and similar to other fibrotic conditions, is characterized by deposition of ECM in heart tissues, which ultimately results in heart failure and death.^143^ A suitable disease model for studying cardiac fibrosis, is induced Diabetic Cardiomyopathy (DbCM). Three distinct studies have revealed the role of different compounds in inhibiting TGF‐β/ SMAD pathway in protection against fibrosis.^144^, ^145^, ^146^ By applying a high‐fat diet and streptozotocin, induction of cardiomyopathy was achieved in all three studies. Matrine has shown anti‐fibrotic properties in hepatic fibrosis,^147^ and in DbCM, it protects against fibrosis and recovers left ventricular (LV) by inhibiting TGF‐β1/R‐Smad signalling pathway, although it does not affect levels of nhibitory Smads like Smad7.^146^ Dapagliflozin (DAPA) acts as sodium‐glucose cotransporter 2 (SGLT2) inhibitor, and by reversing endothelial to mesenchymal transition (EndMT), it reverses fibrotic features and indexes.^145^ Additionally, DAPA is capable of impeding TGF‐β/Smad signalling pathway via AMPKα^145^ (Figure 2C). Finally, Empaglifozin (EMP), which is also a SGLT2 inhibitor, inhibits TGF‐β/ SMAD pathway and on the other hand, contributes to protective effects in cardiac fibrosis by activating Nrf2/ARE signalling.^144^ Other components and their related mechanism in protection against cardiac fibrosis are listed in Table 5.

**TABLE 5:** Cardiac fibrosis.

Diseases	Treatment	Model of study	Cell lines	TGF‐β/SMAD	Targets	Observations	Ref
Cardiac fibrosis	Pioglitazone (PIO)	Male C57BL/6 mice; PIO (2.5 mg/kg)	HUVEC PIO (20 μM)	Inhibit	α‐SMA, Vimentin, Col‐I/ III, FN, Smad‐2/3, Twist1, Snail‐1/2,	Treatment with PIO could act against cardiac fibrosis induced by pressure overload.	^148^
Diabetic cardiomyopathy (DbCM)	Matrine	Male and female SD rats; matrine (300 mg/kg)	Cardiac fibroblasts (CFs); 0–2.5 mmol/L	Inhibit	Smad‐2/3/7, Col‐I	In an animal model of DbCM, treatment with matrine by suppressing the mentioned pathway could act against cardiac fibrosis	^146^
DbCM	Dapagliflozin (DAPA)	Male SD rats DAPA (1 mg/kg)	HUVECs DAPA (0–1 μM)	Inhibit	Col‐I/III, Vimentin, α‐SMA, Smad‐4, AMPKα,	Treatment with DAPA could act against by DbCM‐induced cardiac fibrosis	^145^
DbCM	Empaglifozin (EMP)	KK‐Ay mice; EMP (10 mg/kg)	–	Inhibit	α‐SMA, Col‐1/III, Smad‐2/3/7, Nrf2, HO‐1	Treatment with EMP (an inhibitor of sodium‐glucose cotransporter 2) could act against DbCM‐induced cardiac fibrosis	^144^
Myocardial Infarction (MI)	Ganoderma lucidum polysaccharide peptide (GLPP)	Male C57BL/6 mice; 150 mg/kg	Rat neonatal cardiac fibroblasts; GLPP (100 μg/mL)	Inhibit	α‐SMA, Col‐I/III, NOX4, Smad‐2/3	Treatment with GLPP could act against cardiac fibrosis	^149^
MI	Vitamin D3 + Exercise	Male wistar rats; Vit D3 (10,000 IU/kg)	–	Inhibit	Col‐I/III, Smad‐2/3	Treatment with Vit. D3 + exercise training could act against isoproterenol (ISO)‐induced cardiac fibrosis	^150^
Myocardial fibrosis	Losartan	Wistar‐Kyoto rats (WKYs); Losartan (20 mg/kg)	–	Inhibit	Col‐I, Smad3	Treatment with Losartan could act against cardiac fibrosis	^151^

### 3.5 Skin fibrosis

Skin fibrosis occurs as a result of different skin related conditions, such as scleroderma and eosinophilic fasciitis.^152^ LG283 is a synthetic compound and is derived from CUR.^153^ After induction of skin fibrosis by BLM in C57BL/6 mice, skin was thickened and fibrotic changes at molecular level was observed.^154^ Treatment with LG283 reduces capillary vessels and softens skin tissues, and also lowers α‐SMA and phosphorylated Smad3 expressions.^154^

Scleroderma, also known as systemic sclerosis, is connective tissue disorder and is characterized by skin stiffness and vascular abnormalities in internal organs. This disease is categorized as autoimmune, and its exact molecular mechanism is yet to be found.^155^

According to medchemexpress, DZ2002 is an immunosuppressive type III SAHH inhibitor and has beneficial properties for lupus syndrome and systemic sclerosis, only for research purposes. Research has found out that DZ2002 reduces psoriasis‐like skin lesions and inflammation by regulating GATA3 methylation.^156^ Interestingly, DZ2002 has proven to be protective in mice models of systemic sclerosis^157^ (Figure 2D). The mechanism of action involves reduction of TGF‐β‐1 and CTGF, as well as VEGF which is responsible for vascular complications.^157^

Table 6 summarizes application of different compounds for treatment of skin fibrosis.

**TABLE 6:** Skin fibrosis.

Diseases	Treatment	Model of study	Cell lines	TGF‐β/ SMAD	Targets	Observations	Ref
Skin fibrosis	LG283	Female C57BL/6 mice; LG283 (40 and 80 mg/kg)	Normal human dermal fibroblasts, A549; DMSO‐diluted LG283 (0.5 and 4.5 μM)	Inhibit	Snail1/2, α‐SMA, ZEB1/2, COL1A2	Treatment with LG283 could act against skin fibrosis via inhibiting EMT pathways (Snail and TGF‐β/Smad)	^154^
Skin fibrosis	HPH‐15	Female C57BL/6 mice; HPH‐15 (100 mg/kg)	Human dermal fibroblasts; HPH‐15 (10 μM)	Inhibit	α‐SMA, Col1A2, FN1, CTGF, Smad‐3	Treatment with HPH‐15 could act against skin fibrosis induced by TGF‐β	^158^
Corneal wound healing	IGF‐1	–	Primary human corneal keratocytes, 10 nm/mL	Inhibit	Smad‐3/7	Fibrosis in human keratocytes could be mediated via treatment with IGF‐1	^159^
Systemic sclerosis	DZ2002	C57BL/6 mice; DZ2002 (50 and 100 mg/kg)	Human dermal fibroblast; DZ2002 (0–200 μM)	Inhibit	α‐SMA, VEGF ICAM‐1, VCAM‐1, Smad‐3/4/7,	Treatment with DZ2002 could act against BLM‐induced systemic sclerosis	^157^

### 3.6 Other fibrotic conditions

In addition to the more common cases of fibrosis mentioned earlier, there are also some uncommon instances that have not been extensively researched. Table 6 contains four distinct conditions, including Muscle Fibrosis, Choroidal Neovascular Fibrosis (CNV), Proliferative Vitreoretinopathy (PVR) and Epidural Fibrosis.

Muscle fibrosis usually occurs in dystrophies especially Duchenne muscular dystrophy (DMD) and effects a significant portion of patients. In other cases such as muscle injuries, muscle fibrosis rarely occurs.^160^

CNV refers to formation of new blood vessels in choroid layer of the eye and could lead to vision loss. Risk of this condition increases with age, and is more prevalent in individuals over the age of 75.^161^

PVR is a complication that can occur after a person has undergone surgery for a retinal detachment. It is characterized by the growth of abnormal tissue on the surface of the retina, which can cause the retina to detach again.^162^

Presented in some cases lumbar spinal surgery patients, Epidural Fibrosis causes a great pain. It has been shown that Scar formation and excessive ECM in epidural space is the main cause.^163^

All of these four conditions are studied in vivo, and it has been shown that except for muscle fibrosis, Pirfenidone (PFD) for CNV, Artesunate (ART) for PVR and Taurine (Tau) for Epidural Fibrosis could act as anti TGF‐β/ SMAD and they have the potential to ameliorate these conditions. Table 7 describes the exact molecular mechanisms of these compounds.

**TABLE 7:** Other fibrotic conditions.

Diseases	Treatment	Model of study	Cell lines	TGF‐β/SMAD	Targets	Observations	Ref
Muscle fibrosis	Sulforaphane (SFN)	Male C57BL/10ScSn‐Dmdmdx/NJU mice; SFN (2 mg/kg)	–	–	α‐SMA, FN, Smad‐2/3, Col‐I, PAI‐1, TIMP‐1, Nrf‐2	In mdx mice, treatment with SFN by suppressing the Nrf2‐mediated mentioned pathway could act against muscle fibrosis.	^164^
Choroidal neovascular fibrosis (CNV)	Pirfenidone (PFD)	C57BL/6J male mice, PFD (1 μL)	–	Inhibit	Col‐I, α‐SMA, Smad 2/3	Treatment with PFD could act against CNV.	^165^
Proliferative vitreoretinopathy (PVR)	Artesunate (ART)	Pigmented rabbits; ART (20 μg/mL)	ARPE‐19; ART (0–200 μM)	Inhibit	Vimentin, Smad‐3	Treatment with ART could act against the development of PVR by inhibiting the mentioned pathway and EMT process.	
Epidural fibrosis	Taurine (Tau)	Male SD rats; Tau (100 mg/kg)	Primary fibroblast cells; Tau (10 μM ‐ 100 mM)	Inhibit	α‐SMA, Col‐III, Smad‐2/3/7, TGFβRI/RII	Treatment with Tau could act against epidural fibrosis induced by laminectomy.	^166^

## 4 DISCUSSION

TGF‐β/SMAD signalling pathway regulates various cellular functions, such as proliferation, differentiation and apoptosis.^167^ However, uncontrolled activation of this pathway leads to the accumulation of ECM compounds, resulting in organ fibrosis.^10^ Organ fibrosis has important health effects leading to loss of organ function, particularly in liver, lung, kidney and skin. Approximately, one third of natural deaths worldwide is attributed to organ fibrosis and subsequent loss of function of mentioned organs.^168^ Several studies have shown that antioxidants can efficiently reverse the effects of TGF‐β/SMAD pathway in fibrosis, thus contributing to the protection against fibrosis. Although several antioxidants belonging to all three classes of antioxidants have modulating effects on the expression and activity of TGF‐β/SMAD pathway, scavengers are mostly appreciated in this regard.

A generalized function of almost all types of antioxidants is reversing the effects of ROS in cells.^14^ By reducing ROS levels, antioxidants contribute to inhibition of fibrosis. Additionally, antioxidants can down‐regulate expression of TGF‐β and its receptors, and also regulate downstream molecules such as Smads, especially Smad7, and ultimately inhibit the mentioned pathway.

Several antioxidants have shown promising effects in the prevention of organ fibrosis. For instance, N‐acetylcysteine (NAC) is a precursor of glutathione, which is an important antioxidant in the body. NAC has been shown to inhibit TGF‐β induced profibrotic responses and inhibit lung fibrosis.^169^ Vitamin E as another antioxidant also has inhibitory effects on TGF‐β/SMAD signalling pathway.^170^

As we concluded in this article, antioxidant therapy has several benefits over other treatment options for fibrosis. Unlike other treatments, which often target specific aspects of fibrosis and are invasive in nature, antioxidants have a wider impact and disrupt multiple pathways involved in fibrosis. Additionally, antioxidants are relatively safe and are considered as a non‐invasive method for treatment, both in vivo and in vitro, making them a great factor for further studies.

In conclusion, antioxidant therapy has shown potential as a way to treat organ fibrosis by interrupting the TGF‐β/SMAD signalling pathway in animal models and cell lines. However, additional research should be conducted in a greater extent and with more precision, to optimize the dosage and evaluate its effectiveness in various stages of fibrosis with different etiologies. Antioxidant therapy could provide a secure and efficient treatment option for fibrosis in future.

## AUTHOR CONTRIBUTIONS

**Soudeh Ghafouri‐Fard:** Validation (equal); writing – original draft (equal). **Arian Askari:** Validation (equal); writing – review and editing (equal). **Hamed Shoorei:** Investigation (equal); methodology (equal). **Mohammad Seify:** Formal analysis (equal); investigation (equal); visualization (equal). **Yeganeh Koohestanidehaghi:** Supervision (equal); visualization (equal). **Bashdar Mahmud Hussen:** Validation (equal); writing – review and editing (equal). **Mohammad Taheri:** Supervision (equal); validation (equal). **Majid Samsami:** Investigation (equal); methodology (equal).

## FUNDING INFORMATION

Not applicable.

## CONFLICT OF INTEREST STATEMENT

The authors declare they have no conflict of interest.

## Data Availability

Not applicable.
